# The Practice of Birth-Control

**Published:** 1921-04-23

**Authors:** 


### THE EDITOR'S LETTER-BOX.

**Correspondence on all subjects is invited, but we cannot in any way be responsible for the opinions expressed by our correspondents, who must give their name and address as a guarantee of good faith, but not necessarily for publication. Correspondents are reminded that brevity of style and conciseness of statement greatly facilitate early insertion.**

### The Practice of Birth-Control.

*To the Editor of* The Hospital.

Sir,—May I take up a little of your valuable space? "A reflection" on the above subject, in April 9th issue of The Hospital interests me greatly, and the question arises in my mind as to whether large families, numbering from eight to a dozen, are of permanent racial advantage?

I know of three brothers who married and had families of ten, eight, and eleven respectively.

In the eldest family three died in infancy; one was killed in the war.

Of the remaining six (two boys and four girls), one only the eldest son, has married (and that more than twelve years ago), there is no issue!

Three of the girls are earning their living; one is an invalid. All are more or less delicate.

The remaining boy has a conscience, and hesitates to marry, owing to a certain delicacy, and the prospect of one, and probably two, sisters to support later on.

Of the second family of eight, all boys, who lived to grow up, two were killed (one, a married man, leaving a girl). Three of the remaining six are married and two have no children, while one has one boy.

In the third and youngest family all are living with the exception of one boy killed in the war. The eldest (a girl) is at the present time thirty-six, and the youngest (a boy) just eighteen years.

Of the ten living (five boys and five girls) the two eldest boys are married. The eldest has two children (one boy and one girl), the result of eight years of married life. The second boy has been married scarcely a year; no child up to the present. The next two boys are of marriageable age, but for financial reasons will have to wait till late before marrying (if they marry at all). The youngest is still at school. Four of the girls are trained (two teachers and two nurses), the fifth is in training. They may marry, but I doubt it. Should they do so, however, of one thing I am certain, it would be a physical impossibility for any one of them to bear many, and in one or two cases any, children. Why? for one reason, their energies have largely been spent in helping each other, and—in their profession.

Granted they are living happy and useful lives; yes, useful to their generation. *What of the race?*

I am of opinion that very large families, in any grade of society, are a pernicious form of "race suicide."

The reasons are far graver than mere selfishness. Surely some practice of "birth-control," or moderation in reproduction, would in the end be to the advantage of the race, from the point of view of "quantity," as well as quality. It would be interesting to know the opinion of some of your readers.—I am, Sir,

One of a Large Family.

